# Time perspective, control, and affect mediate the relation between regulatory mode and procrastination

**DOI:** 10.1371/journal.pone.0207912

**Published:** 2018-12-10

**Authors:** Eunice E. Hang Choy, Him Cheung

**Affiliations:** 1 Department of Applied Social Sciences, The Hong Kong Polytechnic University, Hong Kong, China; 2 Department of Psychology, The Education University of Hong Kong, Hong Kong, China; Western Sydney University, AUSTRALIA

## Abstract

This study examines the roles of time perspective, affect, and locus of control in mediating the relationship between regulatory mode and procrastination. Participants filled out the Zimbardo Time Perspective Inventory, Positive and Negative Affect Schedule Scale, Multidimensional Locus of Control Inventory, Locomotion and Assessment Scale, and Lay’s General Procrastination scale. Results showed that procrastination was negatively related to locomotion orientation but positively associated with assessment orientation. The relations between regulatory mode and procrastination were mediated by negative affective state, internal sense of control, and negative past and future time perspectives. These findings suggest not only a *behavioral* link between regulatory mode and procrastination but also *affective* and *cognitive* differences in locomotion and assessment orientations that may account for such linkage. The present results also provide empirical support for the theory of locomotion-temporality interface (Kruglanski, Pierro, & Higgins, 2016).

## Introduction

### Overview

Procrastination, or self-regulatory failure to initiate an act despite knowing the negative consequences [1], is a common concern in academics, work, health-related practices, life-long planning, as well as environmental preservations [2–5]. Looking at academic activities alone, 30% to 60% of college students reported procrastination in completing written and reading assignments and preparing for examinations [6]. Many research studies have investigated different external and contextual determinants of procrastination. For example, procrastination is more prominent when the work is monotonous, laborious, or unpleasant [5,7]. Although incentives (e.g., bonus) generally induce early work completion, procrastination is still likely when the gain from task completion is not immediately available [8]; missed opportunities to obtain bonus also increase the likelihood of future procrastination [9].

Procrastination is regarded as an individual trait when it becomes general across situations and stable over time [5, 8]. One line of research has examined *specific* internal factors for procrastination. Specific personality trait, such as neuroticism [10], impulsiveness [1] and lack of conscientiousness [11] have been shown to be strongly linked to procrastination behavior. Another line of research has explored how *general* self-regulatory dimensions [12], such as regulatory mode [13], may be related to procrastination.

Conceivably, culture may be a relevant dimension to consider in investigating procrastination in relation to other trait and personality factors because some previous studies have shown noticeable cross-cultural variations in some of these factors. For instance, Higgins, Pierro, and Kruglanski [14] compared the regulatory mode of individuals from nine nations (England, Japan, Korea, India, Italy, Israel, Poland, Spain, and United States) and found relatively high percentages of locomotion-oriented individuals in India, Italy, and Spain, and low percentages of assessment-oriented individuals in Japan and Korea. Cultural differences in time perspective, which has been considered a correlate of regulatory mode and procrastination, were also found [15–16]. For example, Guo, Ji, Spina, and Zhang [17] showed that Chinese and Chinese Canadians in general valued the past more than the future, while the reverse was true for European Canadians. For procrastination itself, nevertheless, more cross-cultural similarities than differences seem to have been considered [18]. In their investigation on regulatory mode, Higgins et al. [14] argue that cross-cultural variations in distributions of individuals having different orientations do not necessarily reflect cross-cultural personality differences. In the current study, we put the cultural concern aside and focus on the level of individual differences within a culture, examining how time perspective, affect, and locus of control may mediate the relationship between regulatory mode and procrastination.

### Regulatory mode

Two self-regulatory functions, namely, assessment and locomotion, have been identified as important determinants of goal-directed behavior [19]. *Assessment* focuses on evaluating options and contrasting alternatives. Individuals with high assessment orientation deliberately search for the best possible choice; they constantly appraise their own and others’ decisions, goals, and attainments against critical standards [19–20]. In planning a vacation, for example, people with a high assessment tendency collect and evaluate all available information about flights, hotels, promotions as well as contingency plans before making actual arrangements. In contrast, *locomotion* focuses on initiating and progressing towards a desired end-state. Individuals with high locomotion orientation are concerned with maintaining steady action from one state to another without distraction [19–20]. In planning a vacation, people with a high locomotion tendency would consider fewer options in planning a trip in order to get the work done quickly; they avoid undue delays in flight and accommodation booking. Simply put, assessment stresses “doing the right thing’ whereas locomotion features “making it happen” [20].

### Regulatory mode and procrastination

Regulatory mode has been found to correlate with procrastination. Specifically, in academic and organizational settings, procrastination has been shown to be positively associated with assessment but negatively associated with locomotion [13, 21]. But what are some factors that may further explain these relations? With reference to the theory of locomotion-temporality interface [22], we argue that time perspective, locus of control, and affect are the three main factors mediating the relationship between procrastination and regulatory mode.

### Theory of locomotion-temporality interface

According to Kruglanski et al. [22], individuals with high locomotion orientations are especially sensitive to the *flow* and *resource* of time compared to those with high assessment orientations. Flow refers to the representation of time on an imaginary path extending from the past to the future and the thinking that time involves psychological movements along such a path. Resource refers to the thinking that time is a possession that needs to be saved and managed. Flow and resource are therefore two particular ways to think about time. In the following sections, we discuss how locomotors and assessors differ in their perception of flow and resource when thinking about time, which may account for their difference in procrastination tendency.

### Flow characteristics and time perspective

How individuals prioritize activities is inextricably intertwined with their time perception. For example, people conceptualize time as flowing continuously from the past to future and schedule their work accordingly in a successive order [23]. Zimbardo and Boyd [24] contend that a time perspective arises when people psychologically categorize their experiences into past, present, and future frames, with either a positive or negative emotional valence. A temporal bias emerges if the individual frequently adopts one particular frame, and it can become a dispositional pattern when the bias is chronic and general across situations. Accordingly, individuals with a predominant past frame seldom take chances; they make choices using their previous experiences. People with a predominant present frame are more concerned with reality than expectation. Among them, those with a hedonistic present focus are pleasure-seekers while those with a fatalistic present focus are more inclined to view their lives as determined by chance and fate, as opposed to their own effort and action. Finally, individuals with a predominant future frame are concerned with the outcome; hence they strive for long-term goal attainment by committing to work and resisting distractions.

A plethora of studies have examined whether present and future time perspectives are linked to procrastination. A low future time focus has been consistently shown to be associated with procrastination [25–27], yet whether a relation between present time perspective and procrastination exists is less clear [28–29]. In a meta-analysis of 14 samples, Sirois [30] has found converging evidence for a moderate negative relationship between procrastination and future temporal orientation, and a small but significant positive relationship between procrastination and present hedonistic orientation.

As postulated by the theory of locomotion-temporality interface, locomotion, with its focus on making changes and moving towards a desired end-goal, should give rise to an orientation toward the future. We therefore hypothesize that high-locomotion individuals are generally future-focused. This may help account for its negative association with procrastination [13]. Conversely, we also hypothesize that assessment, with its focus on making the right decision and evaluation with critical standards, is more related to a focus on the negative past when appraisal of attainments and comparison of alternatives are especially informative to the individual. As a person who is high in assessment orientation is vigilant about errors in their past decision-making, they are more likely to do considerable planning before proceeding further, hence resulting in an increased procrastination tendency.

### Flow characteristics and affect

Procrastinators often attend more to current mood regulation than the consequence of task avoidance. For example, task postponement has been thought to be an emotional tool serving to reduce stress and anxiety, especially when the work is aversive [31–32]. Nevertheless, it is also common for procrastinators to experience negative emotions, such as anxiety and guilt, as task delay persists [33–38]. Indeed, negative affect has been argued to be one cardinal component to procrastination [39–41]. For example, Corkin and his colleagues [39] differentiate “active delay” from procrastination on the basis of a lack of negative affect and irrationality in the former.

Recent research has shown that affective states are an antecedent of procrastination, not a mere outcome. For example, Ferrari and Díaz-Morales [42] demonstrated that people who procrastinated tended to link their self-concept to task performance and completion, and were more likely to perceive themselves as not being liked by others. Self-generated stress has been found to increase when individuals are preoccupied with past work delay and personal mistakes [43]. Sirois and Giguère [7] followed a group of participants in health-related changes longitudinally and found a link between a low level of positive affect and procrastination.

According to the theory of locomotion-temporality interface, locomotion should be linked to positive affect in general. As locomotion is all about moving away from the past and present and approaching a desired goal in the future, locomotors should show minimal tendency to experience guilt or regret for their wrong-doings or bad decisions in the past, but a strong tendency to experience reconciliation in unsatisfactory events that have taken place. For example, when individuals realize that they have made a wrong purchasing decision, high locomotors are shown to experience less regret than low locomotors [44]. In examining the relationship between the assessment orientation and affect, Kruglanski and his colleagues [20, 45] have linked the theory of objective self-awareness [46] to assessment orientation and suggested that discrepancies between the desired and actual self may lower emotional stability and self-esteem because the self is often under critical evaluation. For example, assessment scores have been shown to be positively associated with the conscientiousness personality trait and depression, and negatively associated with self-esteem in previous American and Italian samples [47].

One purpose of the present study is to examine whether emotion may shape the relations between regulatory mode and procrastination. We hypothesize that individuals with a high assessment focus would experience a high level of negative emotions or a low level of positive emotions. In contrast, as locomotion is less concerned with appraisal against critical standards and more concerned with making progress, high locomotors should experience higher emotional stability and self-esteem [20]. We therefore hypothesize that people with a high level of locomotion tendency would experience a relatively low level of negative emotions or a relatively high level of positive emotions.

### Resource characteristics and locus of control

Another crucial factor for task prioritizing and scheduling is one’s perceived control over the initiation and maintenance of a task and the perceived importance of such control. Since procrastination is regarded as failure in self-management [48], the extent to which people perceive themselves as being able to control their regulation in work may also affect their procrastination tendency. Locus of control (LOC) refers to the degree to which one views an outcome as contingent on internal (e.g., stable personal characteristics and effort) versus external forces (e.g., fate, chance, and powerful others) [49–52]. A body of research has indicated an association between locus of control and procrastination. For example, participants with a high internal locus of control were less likely to procrastinate, and those who procrastinated were shown to have a high external locus of control [53–59]. Locus of control has also been found to moderate the relations between task autonomy and procrastination [60].

The present study examines whether individuals with high assessment and locomotion motivations also display distinctive patterns of locus of control. We seek to explore how the type and degree of control may mediate the relationship between regulatory mode and procrastination. In relation to the concept of resource, the theory of locomotion-temporality interface postulates that time is often conceived as a limited resource (e.g., “life is short”). Given that locomotors are inclined to accomplish goals by transiting from state to state in a steady and continuous manner, they should strive to maximize the use of time by efficient time management. For example, Amato, Pierro, Chirumbolo, and Pica [61] demonstrated that locomotion was positively associated with various measures of time management tendencies (e.g., setting goals, priorities, and preferences for organization). More interestingly, they also found that high locomotors were more likely to have high perceived control of time. Therefore, we hypothesize that people who are high on the locomotion orientation should show a high level of internal locus of control, because the initiation and maintenance of a task is largely determined by one’s internal characteristics such as effort and will power. In contrast, the assessment orientation is about comparing as many alternatives as possible and selecting the best options; this typically involves gathering and evaluating much information about external, environmental factors which are relatively less subject to personal control. Because assessment highlights the importance of external information, we hypothesize that individuals with a high assessment tendency would also tend to have an external locus of control.

### The current study

To our knowledge, most previous studies on regulatory mode have only examined its relation with behavioral procrastination. Not much attention has been paid to plausible cognitive and emotional correlates of regulatory mode that may contribute to the linkage. To fill this gap in the literature, the current study first attempts to replicate the past finding that regulatory mode and procrastination are correlated in university undergraduates. As in some previous studies [61], it is hypothesized that assessment orientation is positively linked to procrastination tendencies whereas locomotion orientation is negatively associated with them. Second, the present study examines some underlying cognitive and affective differences between assessment and locomotion. Specifically, locomotion is hypothesized to be positively related to future time perspective and internal locus of control, but negatively related to negative affect. Assessment is hypothesized to be positively related to negative past perspective, external locus of control, and negative affect. Finally, the current study explores whether these cognitive and affective factors may affect the association between regulatory mode and procrastination. For the linkage between assessment orientation and procrastination, negative past time perspective, external locus of control, and negative affect are assumed to be significantly mediators. For the linkage between locomotion orientation and procrastination, future time perspective, internal locus of control, and a lack of negative affect are hypothesized to be significantly mediators.

The linkages hypothesized in the present study together constitute a theoretical model that is entirely consistent with the theory of locomotion-temporality interface. Outcome from this study therefore complements the theory by extending it along the dimensions of affect, control, and procrastination, using time perspective as the pivot. Results from the present study also provide useful practical information for the management profession, that affect and perception of time are important factors to consider for a better understanding of procrastination behavior.

## Materials and methods

### Participants

A total of 196 undergraduates (114 females) from a university in Hong Kong with a mean age of 19.71 years (*SD* = 0.73 year) were recruited using convenience sampling. Announcements were made in psychology classes and advertisements were put up in the psychology department inviting participation. It was made clear in all these communications that participants would receive psychology course credits for their participation, and no monetary nor any other kinds of reward would be given. Since other studies were also available for sign-up to fulfill course credit requirement, participation in this particular study was seen as voluntary. All the recruited participants were fluent Cantonese-English bilinguals with normal hearing and normal or corrected-to-normal vision. The research was approved by the Human Subjects Ethics Sub-committee (HSESC) (or its Delegate) of The Hong Kong Polytechnic University before data collection. All procedures involving human participants were in accordance with the ethical standards of the institutional and/or national research committee and with the 1964 Helsinki declaration and its later amendments or comparable ethical standards.

### Procedure and instruments

The participants responded to the Regulatory Mode Questionnaire (RMQ) [20], Zimbardo Time Perspective Inventory (ZTPI) [24], Lay’s General Procrastination Scale (GPS) [62], Multidimensional Locus of Control Inventory [49] and Positive and Negative Affect Schedule Scale (PANAS) [63]. All the scales were presented to each respondent in a random order. Because the participants were fluent users of English as a second language, we were able to administer to them the original English versions of all the scales.

#### Regulatory mode questionnaire

The Regulatory Mode Questionnaire is a 30-item instrument assessing the two regulatory orientations with 12 items for each orientation. In addition there are 6 items for lie detection but they were not used in the present study. Hence the present participants responded to only 24 items. They were asked to judge to what extent each item described them on a 6-point Likert scale (1 = strongly disagree; 6 = strongly agree). The items reflect either assessment (e.g., “I often critique work done by myself and others”) or locomotive tendencies (e.g., “By the time I accomplish a task, I already have the next one in mind”). Five reverse-scored items are included. The scale had good reliabilities: Alpha reliability coefficients for the locomotion and assessment scales from the present sample were.79 and .87, respectively. Two composite scores were computed by averaging the responses separately for locomotion and assessment, with a higher value reflecting a greater tendency for each mode.

#### Zimbardo time perspective inventory

The Zimbardo Time Perspective Inventory consists of 56 self-report statements designed to tap respondents’ orientations towards various temporal frames. The participants were asked to rate to what extent each statement correctly described them (e.g., “It gives me pleasure to think about my past.”) on a 5-point Likert scale (1 = very untrue; 5 = very true). The inventory has 5 subscales: past-negative, past-positive, present-fatalistic, present-hedonistic, and future time perspectives, and includes 5 reverse-scored items. Alpha reliability coefficients for the subscales from the present sample ranged from .80 to .89. A composite score for each subscale was computed by averaging the responses for each frame, with higher scores reflecting stronger orientations towards a temporal frame.

#### Lay’s general procrastination scale

Lay’s General Procrastination Scale is a 20-item measure designed to tap trait procrastination across different situations. The participants reported their agreement with each statement such as “In preparing for some deadline, I often waste time by doing other things.” Ratings were made on a 5-point Likert scale ranging from 1 (extremely uncharacteristic) to 5 (extremely characteristic). The alpha reliability coefficient from the present sample was .92. The scale has 10 reverse-scored items. A composite score was computed by summing up all the scores for each item, with a higher value reflecting a stronger procrastination trait.

#### Multidimensional locus of control inventory

The Multidimensional Locus of Control Inventory consists of three subscales designed to measure locus of control: Internal Scale, Powerful Others Scale, and Chance Scale. Each subscale consists of 8 items. The inventory taps the extent to which one believes that event outcomes are affected by internal forces (e.g., “I can pretty much determine what will happen in my life”), powerful others (e.g., “Getting what I want requires pleasing those people above me”), and chance (e.g., “To a great extent my life is controlled by accidental happenings”). Respondents were asked to rate their agreement to each of the statements on a 6-point Likert scale (-3 = disagree strongly; 3 = agree strongly). The alpha reliability coefficients for the subscales from the present sample ranged from .83 to .89. Three composite scores were computed by adding up the responses for each of the three subscales.

#### Positive and negative affect schedule scale

The Positive and Negative Affect Schedule Scale is a 20-item instrument that taps the feelings respondents have experienced during the week of their participation. It includes a positive and a negative affect scale, each consisting of 10 items. On a 5-point Likert scale (1 = very slightly or not at all; 5 = extremely), the participants rated the extent to which each word in the scale described their emotions or feelings. The scale had good reliabilities from the present sample (alpha reliability coefficients: .92 for the Negative Affect Scale and .96 for the Positive Affect Scale). Two composite scores were computed by summing the responses separately for the positive and negative scale, with higher scores indicating stronger affects.

## Results

### Regulatory mode and procrastination

Results from the present study were consistent with those from previous research on the relation between regulatory mode and procrastination. Locomotion was negatively related to procrastination (*r(194)* = -.53; *p* < .001), while assessment was positively related to procrastination (*r(194)* = .26; *p* < .001).

### Cognitive and affective differences in regulatory mode and procrastination

Descriptive statistics and bivariate correlations are shown in Tables 1 and 2 respectively.

**Table 1:** Descriptive statistics.

	Range	*M*	*SD*
1. Procrastination	20–100	52.72	13.55
2. Locomotion	1–6	3.99	.64
3. Assessment	1–6	3.58	.73
4. Past positive	1–5	3.48	.65
5. Past negative	1–5	3.22	.78
6. Present hedonistic	1–5	3.29	.58
7. Present fatalistic	1–5	2.98	.75
8. Future	1–5	3.47	.51
9. Internal control	0–48	31.91	8.00
10. Powerful others	0–48	23.58	10.47
11. Chance	0–48	23.85	9.96
12. Positive affect	10–50	31.01	9.18
13. Negative affect	10–50	18.22	9.29

**Table 2:** Bivariate correlations.

	1	2	3	4	5	6	7	8	9	10	11	12
1. Procrastination	-											
2. Locomotion	-.53**	-										
3. Assessment	.26**	.06	-									
4. Past positive	-.34**	.49**	-.10	-								
5. Past negative	.37**	-.12	.63**	-.21**	-							
6. Present hedonistic	.08	.32**	.32**	.37**	.39**	-						
7. Present fatalistic	.45**	-.15*	.41**	-.04	.66**	.57**	-					
8. Future	-.62**	.71**	-.12	.45**	-.01	-15*	-.24**	-				
9. Internal control	-.41**	.53**	-.02	.48**	-.01	.41**	-.02	.54**	-			
10. Powerful others	.33**	-.08	.53**	-.11	.61**	.40**	-.60**	-.03	.20**	-		
11. Chance	.40**	-.09	.50**	-.09	.63**	.49**	.79**	-.13	.14	.75**	-	
12. Positive affect	-.23**	.64**	.01	.42**	-.11	.47**	.05	.36**	.46** 46	.06	.08	-
13. Negative affect	.44**	-.20**	.33**	-.18*	-.44**	.20**	.42**	-.22**	-.09	.44**	.45**	.10

Note. *N* = 196

* *p* < .05

** *p* < .001

As hypothesized, locomotion tendency was positively related to future time perspective (*r(194)* = .71; *p* < .001), internal locus of control (*r(194)* = .53; *p* < .001), and negatively related to negative affect (*r(194)* = -.20; *p* < .001). Assessment was positively related to negative past perspective (*r(194)* = .63; *p* < .001), external locus of control (*r(194)* = .53; *p* < .001 for the Power Others score and *r(194)* = .50; *p* < .001 for the Chance score), and negative emotion (*r(194)* = .33; *p* < .001).

Procrastination was also significantly correlated with time perspective (past-negative, past-positive, present-fatalistic, and future), locus of control (internal, powerful others, and chance), and affect (positive and negative) (see Table 2).

### Multiple regression analyses: Regulatory mode, cognitive and affective differences, and procrastination

Multiple regressions were performed to test the relations between regulatory mode and procrastination. As some previous studies showed that male students were more likely to procrastinate than their female counterparts [64–67], gender was entered together with age into the first block as control variables in the analyses. Assessment and locomotion orientations were entered into the second block; time perspective (past-negative, past-positive, present-fatalistic, present-hedonistic, and future), locus of control (internal, powerful others, and chance), and affect (positive and negative) were entered into the last block. Regression results are shown in Table 3.

**Table 3:** Regression results: Unique contributions of predictors in each block with procrastination as outcome variable.

Predictor	β	Δ*R*^*2*^	Δ*F*	*df*
Block 1		.04	4.41*	2, 193
Gender	-.20*			
Age	.06			
Block 2		.33	51.24**	2, 191
Assessment	.28**			
Locomotion	-.53**			
Block 3		.21	9.04**	10, 181
Past-negative	.16*			
Past-positive	.07			
Present-fatalistic	-.02			
Present-hedonistic	.04			
Future	-.44**			
Internal locus of control	-.17*			
Powerful others	.05			
Chance	.10			
Positive affect	.05			
Negative affect	.14*			

Note. *N* = 196

* *p* < .05

** *p* < .001

Consistent with previous findings, there was a significant gender effect on procrastination tendency, with men (*M* = 55.93, *SD* = 10.02) receiving higher scores than women (*M* = 50.41, *SD* = 15.22). After controlling for age and gender, in block 2, locomotion orientation was negatively associated with procrastination whereas assessment orientation was positively associated with it. The second model as a whole (i.e., block 1 + block 2) explained 37.8% of variance in procrastination (*F*(4, 191) = 28.97, *p* < .001, *R*^*2*^ = .38, adjusted *R*^*2*^ = .37).

The full model, i.e., block 1 + block 2 + block 3, explained a significant proportion of variance in procrastination (*F*(14, 181) = 18.22, *p* < .001, *R*^*2*^ = .59, adjusted *R*^*2*^ = .55). The associations between regulatory modes and procrastination were however not significant in this final model, in which past-negative perspective, future perspective, internal locus of control, and negative affect were shown to be significantly linked to procrastination.

### Multiple mediation analyses: Effects of cognitive and affective factors on regulatory mode and procrastination

**Fig 1:**
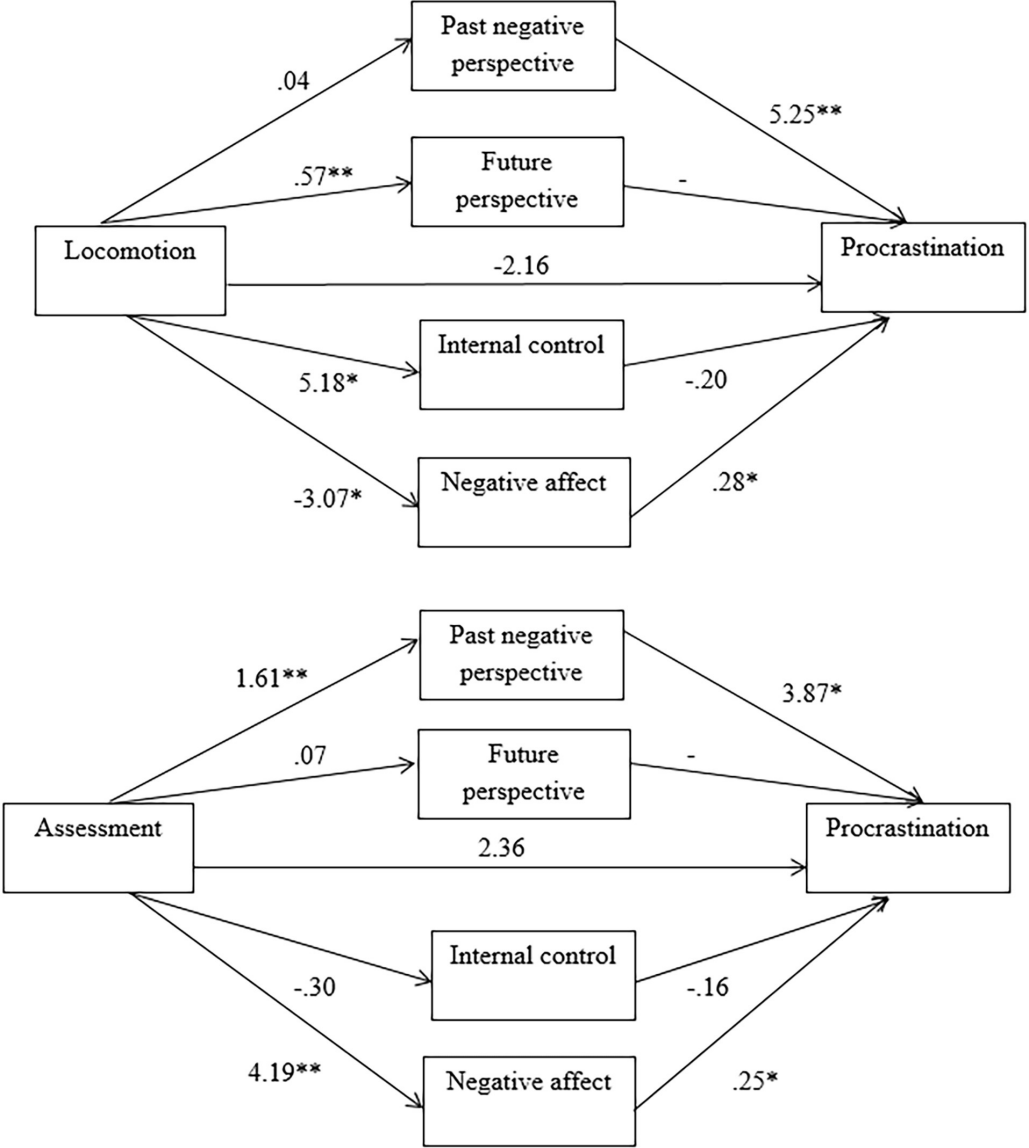
Multiple mediation models for assessment and locomotion. The coefficients represent the effects of regulatory modes on the mediators and procrastination. * *p* < .05 ** *p* < .001.

Multiple mediation analyses were then conducted to examine the roles of past-negative and future time perspectives, internal locus of control, and negative affect in the associations between regulatory modes and procrastination. Two multiple mediation models, one for assessment and one for locomotion, were analyzed using bootstrapping procedures with bias-corrected confidence estimates [68–70]. Using the PROCESS macro for SPSS as developed by Preacher and Hayes [70], 95 percent confidence intervals were obtained with 5,000 bootstrapping resamples. Fig 1 summarizes the multiple mediation models.

Procrastination was regressed on locomotion tendency, with past-negative perspective, future perspective, internal locus of control, and negative affect as mediators; gender was also entered as a covariate in the model. First, the analysis showed that the *total effect* of locomotion orientation on procrastination was significant (β = -10.63, *t*(188) = -7.45, *p* < .001, confidence interval [CI] = -13.46 to -7.82). Second, the *direct effect* of locomotion tendency on procrastination was not significant in the presence of the mediators (β = -2.16, *t*(188) = -1.37, *p* = .17, CI = -5.27 to .95). Note that locomotion tendency was a significant predictor without controlling for the mediators. Further, the *total indirect effect* of locomotion orientation on procrastination was significant (β = -8.48, *p* < .001, CI = -11.84 to -5.27, not containing zero). Overall, the multiple mediation model was significant (*F*(6, 188) = 27.89, *p* < .001, *R*^*2*^ = .56, adjusted *R*^*2*^ = .55).

Procrastination was also regressed on assessment tendency with the same mediators and covariate. The *total effect* of assessment tendency on procrastination was significant (β = 5.17, *t*(188) = 4.81, *p* < .001, CI = 3.05 to 7.28). In the presence of the mediators, the *direct effect* of assessment orientation on procrastination was non-significant (β = 2.37, *t*(188) = 1.95, *p* = .05, CI = -.03 to 4.76). Note that it was a significant predictor when the mediators were not controlled for. The *total indirect effect* of assessment orientation on procrastination was significant (β = 2.80, *p* < .001, CI = .30 to 5.34, not containing zero). Overall, the multiple mediation model was significant (*F*(6, 188) = 44.86, *p* < .001, *R*^*2*^ = .57, adjusted *R*^*2*^ = .55).

In sum, both locomotion and assessment were significantly associated with procrastination; time perspectives, sense of control, and affect as mediators helped explain why there was a relationship between regulatory mode and procrastination. Specifically, individuals who were high in locomotion or assessment differed in their past and future temporal foci, internal sense of control, and level of negative affect, which in turn affected their procrastination tendencies.

## Discussion

Regulatory mode was examined in relation to procrastination in the present study. Locomotion, with an emphasis on moving from state to state and getting things done, was found to be negatively associated with procrastination. On the other hand, assessment which focuses on evaluating alternatives and selecting the best possible option was shown to be positively related to procrastination. Given the current findings, nevertheless, cautious interpretations should be made with regard to the relationship between regulatory mode and success in goal pursuit in general. These findings do not suggest in any straightforward way that individuals with a high assessment or low locomotion orientation are simply inefficient in pursuing their goals. This is because procrastination constitutes only one of the many facets of the relation between regulatory mode and goal pursuit.

The present findings not only complement past research by providing further support for a relation between regulatory modes and the behavioral aspect of procrastination, but also extend previous conclusions by highlighting the underlying cognitive and affective factors that mediate the relation. *Cognitively*, the participants who had high locomotion orientation showed a temporal focus in the past and especially in the future; the participants with a high assessment tendency on the other hand showed a particularly prominent time perspective centering on their negative past. Moreover, locomotion was positively associated with internal locus of control, whereas assessment was positively related to an external sense of control (i.e., power others and chance). *Affectively*, the individuals with high locomotion orientation were also more likely to show positive affect while those with high assessment scores were inclined to experiencing negative emotions (see Table 2). Importantly, both cognitive and affective factors mediated the relationship between locomotion and the *behavioral* tendency of procrastination.

This study was however limited by the adoption of a one-time and scale-based measure of the procrastination trait. Multiple behavioral measures of task delay should be more ideal. Specifically how well self-report surveys inform us about actual procrastination behavior remains an open question, and this constitutes a major weakness of the present study. Do studies measuring actual procrastination behavior and those using participant self-report paint similar or different pictures of procrastination and its relationships with other constructs? The problem of method variance also affects how we may interpret findings from the other measures used in this study, since they are all self-report measures. We acknowledge the problem and attempt to search for clues in the literature. For example, while some previous studies have shown that participants’ reported procrastination tendency was unrelated to actual delay in studying [71], others have demonstrated a significant relation between trait-based procrastination and actual academic delay [41, 72]. It has been argued that individuals often evaluate their past behavior in a negative light, and that self-report assessment and actual behavioral observation may show poor convergence [41]. Instead of measuring trait procrastination once as in the aforementioned studies, Krause and Freund [40] recorded both reported procrastination states and actual delayed studying over 16 time points. Their results indicated that self-report procrastination was moderately linked to observed academic delay. In addition, only self-report procrastination predicted negative affective well-being, which was considered an important emotional indicator of procrastination [73–75]. Orehek et al. [21] (Study 5) measured both undergraduates’ self-reported and actual procrastination (assignment submission time), and found that self-reported procrastination tendency significantly correlated with actual submission time. Therefore, while acknowledging that method variance (behavior vs. self-report) constitutes a general problem for data interpretation in the present study, we think that self-report assessments are still quite informative especially in the case of self-reported procrastination, despite some previous concerns over its validity.

Another concern has to do with the fact that the current findings are based on analysis of cross-sectional data all obtained at one time point, and thus the directions of effects are unclear because of the very nature of the data. Cautions should be taken not to make strong assumptions about causal relationships. For instance, it is entirely possible that procrastination tendencies induce affective changes which may in turn influence time perspective. Assumptions about causality based on the present patterns of findings warrant more stringent testing using an experimental design in future research.

Finally, procrastination has been regarded by some as a multidimensional concept [76] and this multidimensionality could motivate interesting further research. For example, two distinct motives, namely avoidant and approach, have been suggested to underlie procrastination [26, 77, 78, 79]. While some individuals procrastinate to seek arousal when rushing to meet a deadline, others procrastinate simply to delay any possible negative feedback that may compromise their self-worth. In view of this, future research that measures both trait and actual task delay, as well as the various motives of procrastination, should help elucidate the dynamic interplay among the cognitive, affective, and behavioral components of regulatory modes in relation to procrastination.

## Supporting information

S1 Dataset RM_procrastination study (Mar 2015 dataset)_for manusrcipt.sav. (SAV)
